# Functional Neuroanatomy and Behavioural Correlates of the Basal Ganglia: Evidence from Lesion Studies

**DOI:** 10.3233/BEN-2012-120264

**Published:** 2013

**Authors:** Peter Ward, Stefano Seri, Andrea Eugenio Cavanna

**Affiliations:** ^1^The Michael Trimble Neuropsychiatry Research GroupDepartment of NeuropsychiatryBSMHFT and University of BirminghamBirminghamUK; ^2^School of Life and Health SciencesAston Brain CentreAston UniversityBirminghamUK; ^3^Department of Motor Neuroscience and Movement DisordersInstitute of Neurology and University College LondonLondonUK

**Keywords:** Basal ganglia, behaviour, cognition, emotion, lesion, neuropathology

## Abstract

*Introduction:* The basal ganglia are interconnected with cortical areas involved in behavioural, cognitive and emotional processes, in addition to movement regulation. Little is known about which of these functions are associated with individual basal ganglia substructures.

*Methods:* Pubmed was searched for literature related to behavioural, cognitive and emotional symptoms associated with focal lesions to basal ganglia structures in humans.

*Results:* Six case-control studies and two case reports were identified as relevant. Lesion sites included the caudate nucleus, putamen and globus pallidus. These were associated with a spectrum of behavioural and cognitive symptoms, including abulia, poor working memory and deficits in emotional recognition.

*Discussion:* It is often difficult to precisely map associations between cognitive, emotional or behavioural functions and particular basal ganglia substructures, due to the non-specific nature of the lesions. However, evidence from lesion studies shows that most symptoms correspond with established non-motor frontal-subcortical circuits.

## 1. Introduction

The basal ganglia are a collection of interconnected subcortical structures located at the base of the forebrain. These structures include the striatum (caudate nucleus, putamen and nucleus accumbens), the globus pallidus (pars interna and pars externa), the substantia nigra (pars compacta and pars reticulata) and the subthalamic nucleus [1].

The basal ganglia receive inputs from many different cortical regions, entering through the striatum. The information is processed via direct and indirect pathways, passing through the globus pallidus pars externa, subthalamic nucleus and substantia nigra pars compacta, and leaves through the pars interna of the globus pallidus and substantia nigra pars reticulata to go to the thalamus. The thalamus feeds back to specific cortical areas, creating a closed feedback loop (frontal-subcortical loop) (Fig. 1) [2]. As such, the basal ganglia are able to integrate information on convergent pathways from large cortical regions and feed back to cortical areas involved in motor planning [3].

Traditionally the basal ganglia have been viewed mainly as structures for regulating voluntary movement, motor planning and procedural learning [4]. These are conducted by two frontal-subcortical loops, the motor and oculomotor circuits. Recent literature

ISSN 0953-4180/13/$27.50 © 2013 – IOS Press and the authors. All rights reserved

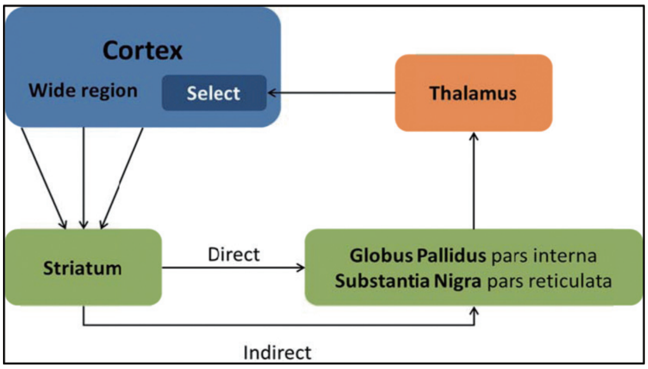

Fig. 1. Frontal-subcortical loop: the basal ganglia integrate information from a wide range of cortical areas, and feed back to selected frontal regions via the thalamus.

however has demonstrated that the basal ganglia are also interconnected with cortical areas involved in cognitive, behavioural and emotional processes [5]. These are mediated by the dorsolateral prefrontal circuit, which regulates executive functions such as planning and working memory, the anterior cingulate circuit, involved in motivation, and the orbitofrontal circuit, which plays a role in selecting the appropriate behavioural response to emotional information [4].

These three loops have the same basic circuit structure, and are closely related anatomically [4]. It is therefore not surprising that the phenotype of movement disorders caused by lesions in the basal ganglia such as Parkinson's disease, Huntington's disease and Tourette's syndrome also include behavioural symptoms (as they affect both the motor and behavioural loops). Patients with Parkinson's disease, which is characterised by the triad of motor symptoms tremor, rigidity and bradykinesia, often present impaired working memory and impulse control as well as affective disorders, reflecting involvement of the non-motor loops [6]. It has been shown that these non-motor disorders can have a larger impact on patients' quality of life than motor symptoms [7].

This paper aims to review the behavioural, cognitive and emotion symptoms caused by focal lesions to basal ganglia substructures in humans. This neuropathological approach helps to understand the function of the basal ganglia, and inform the treatment of neuropsychiatric disorders by targeted therapies such as deep brain stimulation by indicating the implications of lesioning or inhibiting specific basal ganglia substructures.

## 2. Methods

A literature review was conducted on Pubmed using the search terms "focal" "lesion", "basal ganglia" and "behaviour", "affective", "cognitive", "emotional", "executive", "neuropsychological" or "motivation". The search terms were also matched to expanded subject headings. A total of 94 published articles were found. The search was then limited to original studies on human subjects written in the English language. Titles and abstracts were scrutinised for relevance and papers were included if they specified which basal ganglia substructures were affected and excluded if they focused on damage to the frontal cortex or patients had known neurodegenerative disorders (which are likely to cause more widespread damage to the brain). Finally, additional studies were identified by snowballing from the extracted articles

## 3. Results

The search strategy identified only 8 studies (6 case-control studies and 2 case reports) describing patients with focal lesions to basal ganglia substructures and associated behavioural, emotional and cognitive symptoms. All of the studies used MRI to localise lesion sites.

### 3.1. *Case-control studies*

Four of the case-control studies assessed learning and memory deficits in participants, and two assessed the ability of participants to recognise positive and negative emotions in other people.

#### 3.1.1. *Learning and memory*

A study by Ell et al. [8] featured 6 patients with unilateral basal ganglia lesions caused by strokes involving the putamen. The lesion extended into the caudate nucleus in one patient and outside the basal ganglia (to include the white matter, insular cortex or thalamus) in the remaining patients. On neuropsychological examination, when tested against 23 healthy controls, the patients were found to have deficits in working memory. In another task requiring participants to screen out irrelevant information the patients performed similarly to controls.

Bellebaum et al. [9] looked at 11 patients with selective basal ganglia lesions to the putamen. The lesions were caused by cerebrovascular events, haemorrhage in two patients and ischaemic events in the other nine, and were unilateral in ten of the 11 patients. The globus pallidus pars externa was also affected in six of the patients and the caudate nucleus in three. The patients did not have significant deficits in working memory when compared to 13 healthy controls. In a separate task, the patients performed comparably to a group of 18 healthy controls (different from the working memory control group) in learning which buttons to press when shown different symbols to gain a reward. One patient with lesions to the putamen, globus pallidus pars externa and caudate nucleus was better at learning associations than the controls. When the button-symbol associations were changed the patients struggled to learn the new associations. This was particularly noted in patients with lesions to the dorsal striatum.

Ell et al. [10] looked at 7 patients with unilateral lesions caused by stroke. The putamen was affected in all patients. The lesion extended into the caudate nucleus for one patient and into white matter for another patient (the lesion may also have included the insular cortex). Finally two of the patients may have had additional strokes affecting the thalamus. The patients performed less well than 9 healthy controls in the "rule-based" task, where participants performed better if they could discern a set of rules for putting lines into 4 different groups by categorising by their length and then by their orientation (e.g. divide into long and short lines and then sub divide into shallow and steep lines). Patients performed as well as controls in the "information-integration" task where there was no easily discernible pattern between groups of lines and so it was more effective to integrate the lines' length and orientation into single concepts (e.g. short steep, medium shallow, long steep and medium very steep lines).

A study by Pulsipher et al. [11] looked at 73 patients who had been poisoned by carbon monoxide and compared their symptoms to changes in the volumes of basal ganglia structures in at 2 weeks and 6 months following poisoning. The study found that slower mental processing speeds correlated with smaller caudate nucleus volumes at 2 weeks and putamen, globus pallidus and total basal ganglia volumes at 2 weeks and 6 months post poisoning. Verbal memory deficits correlated with smaller putamen, globus pallidus and total basal ganglia volumes at 6 months.

### *3.1.2. Emotional recognition*

Paulmann et al. [12] assessed the ability of 12 patients to detect another person's mood by the tone of their voice (emotional prosody) using electroencephalogram recordings. The patients all had damage to the striatum caused by cerebrovascular events (ischaemic and embolic stroke, haemorrhage, intracerebral bleeding and arterio-arterial infarction). In most of the patients, the lesions also extended into other regions including the thalamus, white matter and parietal operculum. Participants were played audio recordings that conveyed anger, disgust, fear or neutral emotions while recording their brain electrical activity. When compared with 12 healthy controls, the patients were found to have similar changes in the early stages auditory processing, postulated to correspond to the integration of emotional cues. However, late auditory responses, which correspond to the recognition of the emotion conveyed by the cues were impaired in the patient group

Finally a study by Calder et al. [13] compared 4 patients with focal lesions to the ventral striatum (mainly to the putamen) to a control group of 3 patients with dorsal basal ganglia lesions (including the putamen, globus pallidus and caudate nucleus). The insula cortex and internal capsule were also affected in some of the patients. The lesions were caused by cerebrovascular incidents, and there was no overlap of lesion sites between the groups. Participants were asked to recognise emotions (sad, happy, fear, disgust and anger) in audio recordings and from photographs of different facial expressions. The ventral striatum patients were only significantly impaired in recognizing signals of aggression or anger. The control group did not have any significant impairment.

## *3.2. Case reports*

Barbarulo et al. [14] report on a 50 year old male patient who had suffered from an ischaemic lesion to the left basal ganglia due to a stroke, specifically to the head of the caudate nucleus and anterior part of the putamen. Small lesions were also observed in other cortical areas including the left anterior temporal region, the right capsule-nuclear and paraventricular regions. The patient was also reported to have hypoperfusion of left prefrontal areas. On investigation the patient performed poorly on verbal fluency tasks, had frequent perseverations (continuous repetition of a particular action despite the lack of a stimulus) in the Wisconsin Card Sorting Test and found it difficult to control incorrect reactions to stimuli in go-no go tasks. He also presented with planning difficulties in visuo-spatial tasks.

Benke et al. [15] discussed case reports of two patients, PJ (male, aged 56) and AS (female, aged 58), who had suffered from spontaneous left basal ganglia haematoma. In PJ the caudate nucleus was mainly affected, whilst in AS mainly the globus pallidus and putamen were affected with relatively minor damage to the caudate nucleus. There was no cortical damage in either patient. Premorbidly PJ successfully ran a small business and AS was well known for her drive and initiative. However, after the onset of disease both patients were described as being poorly motivated, lacking emotion, initiative or concern for their responsibilities and severely forgetful. PJ was also commonly perseverating. Neither patient exhibited other symptoms typical for depression, but both denied any change to their mental ability or general well-being. On initial examination the patients both suffered from abulia (lack of capacity to make their own decisions or act independently), reduced verbal fluency and word-finding difficulties. They also demonstrated impaired episodic memory, struggled in timed mental arithmetic tasks and had reduced attention and alertness.

## 4. Discussion

This review found that patients with focal lesions to the basal ganglia, specifically the caudate nucleus, putamen and globus pallidus, can present a wide range of behavioural, cognitive and emotional symptoms. These symptoms closely match those known to be associated with the three non-motor frontal subcortical circuits.

Patients tend to present impaired working and verbal memory, slower mental speed and reduced verbal fluency. Some patients present perseveration and difficulty with planning tasks, retain the ability to learn new tasks, but find it difficult to adjust when conditions change. These symptoms are in line with lesions to the dorsolateral prefrontal circuit [16]. Many of the patients included in the reviewed studies had lesions mainly to the putamen, a structure not directly associated with the dorsolateral prefrontal circuit [4]. This could be explained by the lesions often extending into the caudate nucleus, which has been shown to connect to the dorsolateral prefrontal circuit [17] and to contribute to learning functions [18].

Some of the patients also suffered from abulia or a lack of motivation, implying that the anterior cingulate circuit can be affected. This is in concordance with Bhatia and Marsden [19] who also found that lesions to the caudate nucleus (and occasionally putamen and globus pallidus) were associated with abulia.

The orbitofrontal circuit is often associated with behavioural control and social judgment [20]. In this review, patients with lesions to the striatum, more specifically the ventral striatum, had difficulty recognising anger and other negative emotions in another person's face or from the tone of their voice. The patients were thought to be able to integrate information from emotional cues, but then unable to match this to a particular emotion. In Ell et al. [10] patients with basal ganglia lesions were able to integrate information about lines but not go on to recognise patterns between the lines. In both studies the patients could integrate information but not give them any higher associations, implying that lesions to the basal ganglia affect this function. Interestingly these similar dysfunctions occurred in different frontal-subcortical circuits, suggesting that the different circuits perform similar algorithms for different cortical areas.

### *4.1. Limitations*

The main aim of this review was to investigate potential cognitive and behavioural changes in patients with lesions to specific structures within the basal ganglia. Lesions generally involved multiple structures within the basal ganglia, making it difficult to relate one area of the basal ganglia to any specific symptom. The lesions also often extended into structures outside of the basal ganglia, and in some instances included lesions to the frontal cortex. Therefore, non-motor symptoms in patients may not be due to damage to the basal ganglia, but to other parts of the frontal-subcortical loops. This inherent problem of lesional pathological studies could be overcome by interventional techniques, such as deep brain stimulation, where only specific structures are affected by lesions.

Few of the reviewed studies were designed to investigate all the psychological deficits in the patients caused by their lesions to the basal ganglia. The studies generally did not specify which parts of the basal ganglia sub structures were affected; this piece of information is crucial to match lesion sites and symptoms with the different frontal-subcortical circuits which are understood to be restricted to different regions of the same structures [4].

Another limitation of this study is that the coverage of relevant literature is incomplete; some studies have been published in languages other than English and the search was performed on a single database. Extending the search to other databases such as EMBASE, PsycInfo or Thomson Web of Science, and using translation services might have improved the hit ratio Finally, publishing bias may have selected interesting and complex cases which might limit the possibility to generalise these findings

## 5. Conclusion

In conclusion, this report aimed to review the behavioural, cognitive and emotional symptoms caused by focal lesions to basal ganglia substructures in humans. Eight studies were found via a literature search conducted on Pubmed, all of which identified lesions to basal ganglia substructures (specifically the caudate nucleus, putamen and globus pallidus) in patients using MRI scan. Patients suffered from a variety of symptoms including abulia, poor working memory and difficulty recognising emotions in others, corresponding to the three non-motor frontal-subcortical circuits. However as lesions affected multiple structures it was not possible to map particular structures having specific functions. Further work should be done to inform the treatment of neuropsychiatric disorders by focal therapies targeting the basal ganglia, by combining these data with the findings of interventional studies where the lesion site is controlled, such as with deep brain stimulation.
